# Combining electrical stimulation and tissue engineering to treat large bone defects in a rat model

**DOI:** 10.1038/s41598-018-24892-0

**Published:** 2018-04-20

**Authors:** Liudmila Leppik, Han Zhihua, Sahba Mobini, Vishnu Thottakkattumana Parameswaran, Maria Eischen-Loges, Andrei Slavici, Judith Helbing, Lukas Pindur, Karla M. C. Oliveira, Mit B. Bhavsar, Lukasz Hudak, Dirk Henrich, John H. Barker

**Affiliations:** 1 Frankfurt Initiative for Regenerative Medicine, Experimental Orthopedics and Trauma Surgery, J.W. Goethe University, Frankfurt am Main, Germany; 2 J Crayton Pruitt Family Department of Biomedical Engineering, The University of Florida, Gainesville, USA; 3 Laboratory of Experimental Orthopedics, Department of Orthopedics, Justus-Liebig University, Gießen, Germany; 4 Department of Orthopedics, J.W. Goethe University, Friedrichsheim University Hospital, Frankfurt am Main, Germany; 5 Department of Plastic, Reconstructive and Hand Surgery, University Medical Center, Utrecht, The Netherlands; 6 Department of Plastic, Hand and Reconstructive Surgery, BG Trauma Center Frankfurt am Main gGmbH, Frankfurt am Main, Germany; 7 Department of Trauma, Hand and Reconstructive Surgery, J.W. Goethe University, Frankfurt am Main, Germany

## Abstract

Bone Tissue engineering (BTE) has recently been introduced as an alternative to conventional treatments for large non-healing bone defects. BTE approaches mimic autologous bone grafts, by combining cells, scaffold, and growth factors, and have the added benefit of being able to manipulate these constituents to optimize healing. Electrical stimulation (ES) has long been used to successfully treat non-healing fractures and has recently been shown to stimulate bone cells to migrate, proliferate, align, differentiate, and adhere to bio compatible scaffolds, all cell behaviors that could improve BTE treatment outcomes. With the above in mind we performed *in vitro* experiments and demonstrated that exposing Mesenchymal Stem Cells (MSC) + scaffold to ES for 3 weeks resulted in significant increases in osteogenic differentiation. Then in *in vivo* experiments, for the first time, we demonstrated that exposing BTE treated rat femur large defects to ES for 8 weeks, caused improved healing, as indicated by increased bone formation, strength, vessel density, and osteogenic gene expression. Our results demonstrate that ES significantly increases osteogenic differentiation *in vitro* and that this effect is translated into improved healing *in vivo*. These findings support the use of ES to help BTE treatments achieve their full therapeutic potential.

## Introduction

Large non-healing bone defects, secondary to trauma, tumor resection and debridement of non-vital tissue in septic or aseptic non-unions constitute a major challenge for the patients who suffer with them, the physicians who treat them, and the health care systems burdened with their high costs^1–3^. Due to the increased median age of populations in developed countries and the fact that the incidence of non-healing fractures is greater in the elderly this problem is only getting worse^4^. Currently more than 2.2 million bone graft procedures are performed each year to treat bone defects worldwide, costing roughly 2.5 billion US Dollars and posing an enormous financial burden on affected health care systems^3^.

The treatment of choice in these large bone defects is autologous bone grafts^5^, however several other treatments are also used, including allografts, bone graft substitutes, growth factors, distraction osteogenesis, Masquelet induced membrane technique, electrical stimulation and different combinations of these^6–11^. While these treatments provide varying degrees of healing and functional restoration, they can often be lengthy, arduous, and associated with numerous drawbacks and complications.

Bone Tissue engineering (BTE) is an alternative approach, which holds great promise for promoting bone healing and regeneration and overcoming some of the drawbacks of current techniques. Bone tissue engineering approaches generally entail combining bone forming stem cells with scaffolds that restore missing bone volume, and growth factors that control cell-cell and cell-scaffold interactions in the defect. Clinical BTE approaches have demonstrated encouraging early outcomes. In our own clinical trials we have demonstrated safety and feasibility using autologous bone marrow-derived mononuclear cells (BMC) seeded onto β-tricalciumphosphate (β-TCP) in ten patients^12^ and are currently running a phase II trial to demonstrate efficacy (available at: https://clinicaltrials.gov/ct2/show/NCT02803177).

Bone healing is a complex sequence of biological events in which mesenchymal stem cells and bone progenitor cells have been shown to play an important role (reviewed in^13^). Bone marrow derived mesenchymal stem cells (BM-MSC) are the most commonly used adult stem cells in BTE applications, and have been shown to positively influence endochondral ossification and chondrogenesis in *in vitro* and *in vivo* pre-clinical, and clinical studies (reviewed in^14^). In spite of these promising results, low cell number obtained from bone marrow aspirates, donor site morbidity, and diminished multipotent ability of the cells from elderly donors are some of the drawbacks associated with this cell source (reviewed in^15^). Adipose tissue derived mesenchymal stem cells (AT-MSC), on the other hand, have been shown to mimic BM-MSC activity^16^, yet have additional benefits such as, higher cell proliferation, reduced cell senescence, easy accessibility and less invasive harvesting methods. These characteristics together with the important fact that they can be harvested, processed, and delivered in the operating room requiring no second surgery, make AT-MSC a very attractive alternative cell source for clinical use (reviewed in^17^).

Electrical stimulation has been used clinically for more than 40 years to promote bone healing, mostly as an adjunct to standard fracture care^18–20^. The positive effects of direct current electrical stimulation (ES) on bone healing has been widely demonstrated both experimentally^21,22^ and in clinical applications such as, internal and external fixation^23^, delayed or nonunion fractures^24^, osteotomies^25^, bone grafts^26^, and femoral osteonecrosis^27^. We^28,29^ and others^22,30,31^ have shown that low voltage ES, delivered in dosages similar to endogenous electrical fields (10–150 mV/mm), stimulate bone cells to migrate, proliferate, align, differentiate, and adhere to biocompatible scaffolds, all cell behaviors that could contribute to improved BTE treatment outcomes. In addition to effecting these cell behaviors, in *in vitro* studies that exposed osteoblasts and MSC to ES, increased mineralization, extracellular matrix deposition and enhanced expression of the genes such as *Bmp2*, *Bmp4*, *TGF-beta 1* and *ALP* were demonstrated^32,33^. Finally, in a rat limb amputation model we^34^ and others^35–37^ showed that ES stimulates bone growth and the expression of growth factors associated with osteogenesis, suggesting that ES induces cells to produce growth factors through existing pathways. All of the above cell behaviors and functions are associated with enhanced bone healing and when present in BTE-based treatments could provide optimal outcomes. Based on the above we designed the present studies to test if combining ES and BTE treatments would result in optimizing BTE outcomes. To do so we first determined if ES enhanced MSC osteogenic differentiation when the cells were combined with scaffold material in an *in vitro* 3D setup. Then in a rat femur critical size defect model we combined ES and BTE treatments and measured the effect on bone healing.

## Results

### *In vitro*

#### Effect of ES on AT-MSC osteogenic differentiation in 3D culture

AT-MSCs (at a density of 2 × 10^5^ cells/ml) were seeded onto ß-TCP scaffold in osteogenic-supplemented medium in an ES cell culture device and exposed to 100 mV/mm of ES for one hour daily (experimental group) or not (control group).

#### Cell viability

**Figure 1:**
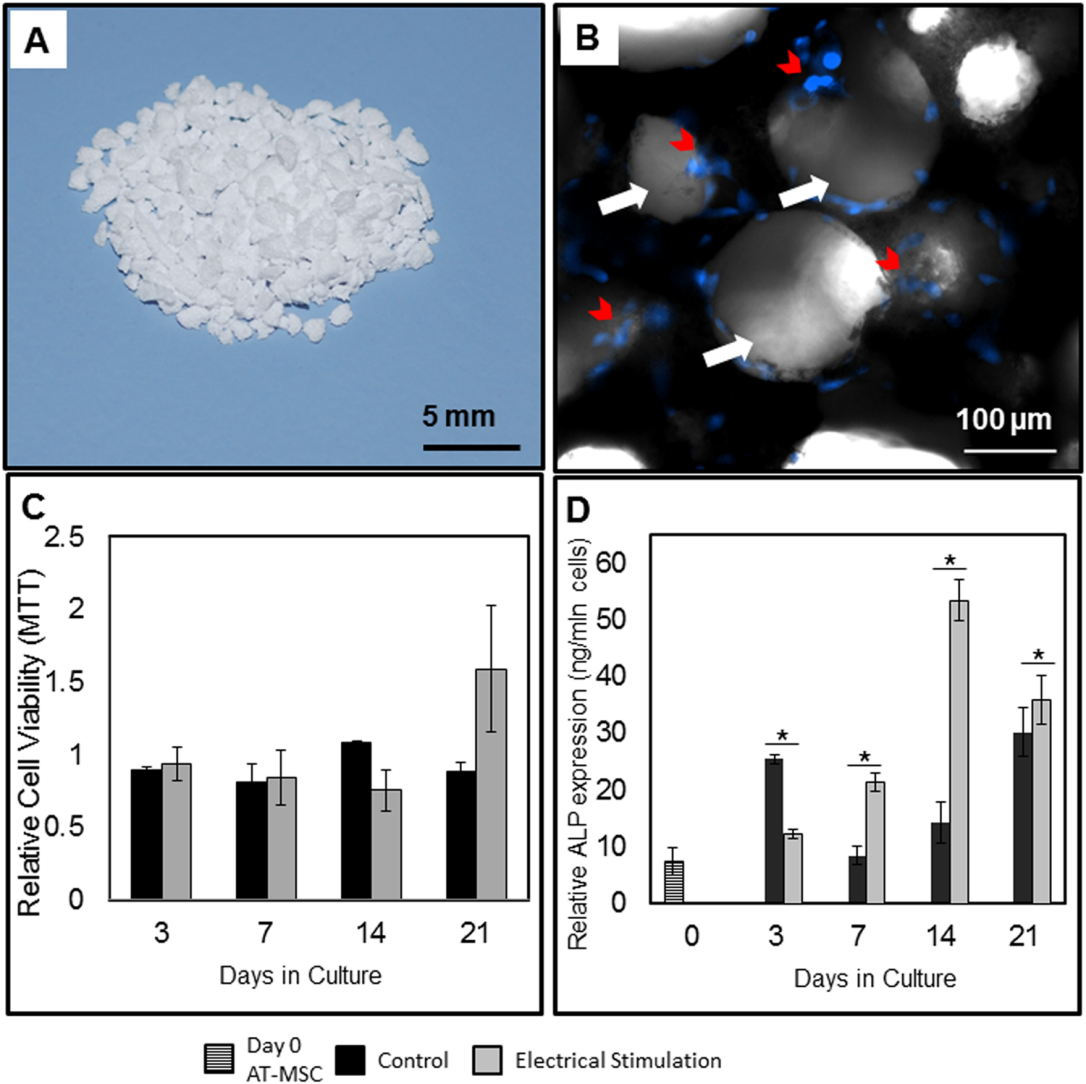
Cell viability and Alkaline Phosphatase activity measurements, *in vitro*. (**A**) ß-TCP scaffold granules. (**B**) AT-MSCs were seeded on ß-TCP granules and stained with DAPI. Red arrows show cell nuclei and black arrows show the pores in the scaffold. (**C**) Effect of electrical stimulation on the viability of AT-MSCs seeded on ß-TCP scaffold, during 21 days in culture. Cell viability measured by MTT assay, shown as fold change relative to day 0. There was no significant difference in viability between cells exposed, and not (controls) exposed to electrical stimulation. (**D**) Effect of electrical stimulation on Alkaline Phosphatase activity of AT-MSCs during 21 days. Data are presented as mean ± SD (n = 3), (*p < 0.05).

DAPI staining was used to visualize cells (nuclei) seeded on ß-TCP scaffold and revealed random cell distribution within the pores and on the surface of the ceramic scaffold material (Fig. 1A). MTT assay showed that ES did not negatively impact cell metabolic activity at any time throughout the 21 day experiment (Fig. 1B).

#### Osteogenic differentiation

Alkaline Phosphatase (ALP) activity measurements showed differing patterns between electrically stimulated and control cells. ALP activity was significantly (p < 0.05) decreased in electrically stimulated AT-MSCs seeded on ß-TCP scaffold at day 3, however, it significantly (p < 0.05) increased at days 7 and 14. No difference in ALP expression was detected between electrically stimulated and control cells at 21 days (Fig. 1C).

#### Gene expression

**Figure 2:**
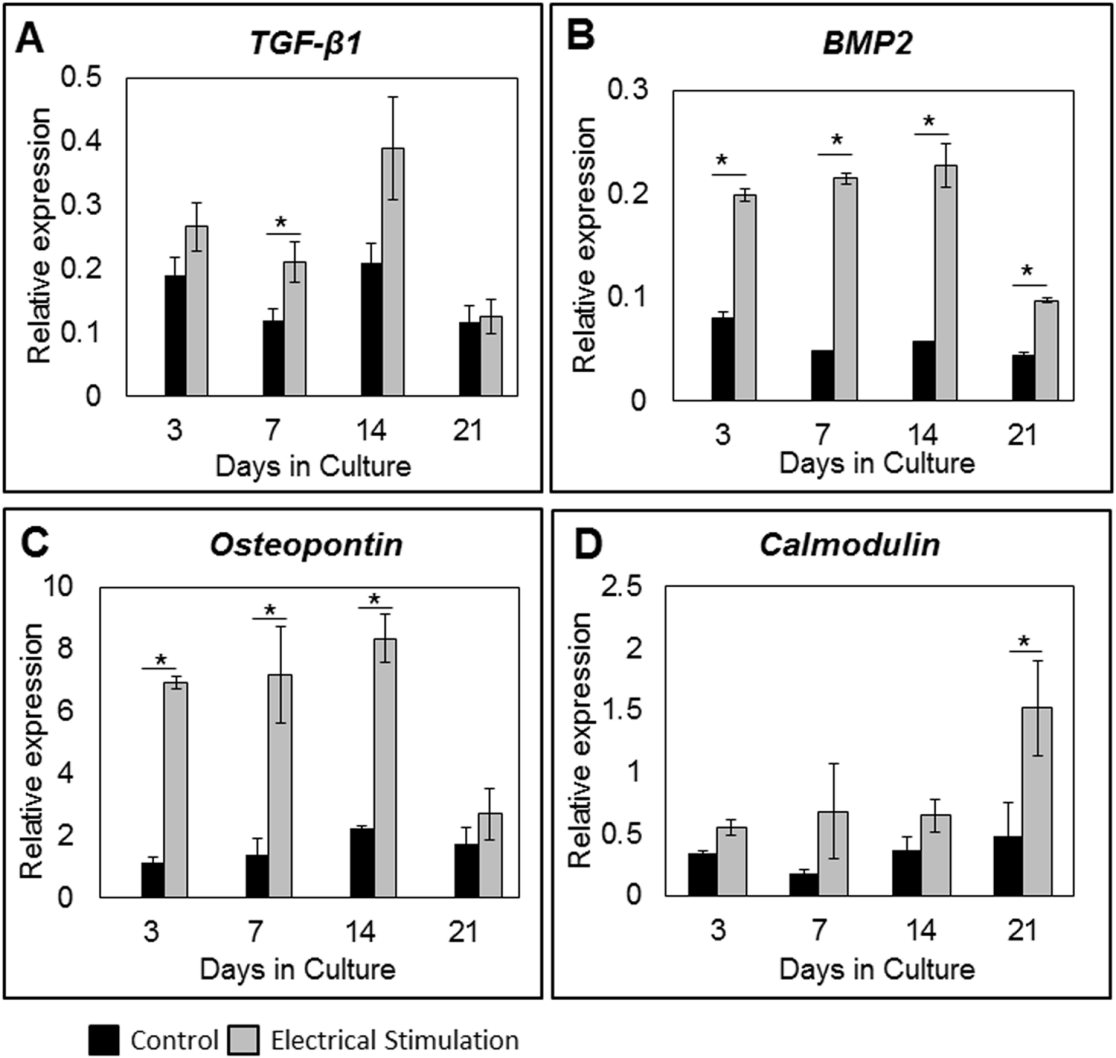
Osteogenic marker gene expression, *in vitro*. Expression of (**A**) *TGF-β1*, (**B**) *BMP2*, (**C**) *Osteopontin*, and (**D**) *Calmodulin* were measured by qRT-PCR and compared between electrically stimulated and control groups. Relative expressions were normalized to *RPLP1* and *YWHAZ* (housekeeping genes). In the experimental group expression of genes *TGF-ß1* (day 7), *BMP2* (days 3, 7, 14 and 21), *Osteopontin* (days 3, 7 and 14), and *Calmodulin* (day 21) was significantly increased in comparison to controls. Data are presented as mean ± SD (n = 3), (*p < 0.05).

Expression of genes that play a role in osteogenesis and bone healing, were measured by qRT-PCR, and showed significant time-dependent changes of *TGF-ß1*, *Bmp2*, *Osteopontin* and *Calmodulin* in electrically stimulated versus control cells (Fig. 2). Expression of growth factor *TGF-ß1* was significantly (p < 0.05) increased in the electrically stimulated group at day 7 (Fig. 2A), while *Bmp2* gene expression was higher in this group at all the time points measured (Fig. 2B), with maximum expression achieved at day 14. Expression of *Osteopontin* was increased 12 fold in the electrically stimulated group on day 3, and this effect continued until day 21 (Fig. 2C). Expression of the *Calmodulin* gene was increased in the electrically stimulated group, reaching a maximum at day 21 (Fig. 2D). No significant difference in expression of osteogenic markers *RunX2*, *Osterix*, *ColIa2* and growth factor *VEGF* were detected between electrically stimulated and control cells at any of the time points (Figure S1).

### *In vivo*

#### Effect of ES on BTE treated rat femur critical size defects

No animals died during surgery, however one rat in the control group died in the immediate postoperative period due to anesthesia associated complications. No abnormal behavior was detected in daily postoperative monitoring of the animals; however at the time femurs were harvested, signs of infection were detected at the defect site in three rats (1 control and 2 sham animals). In 2 rats, in the experimental group, bone fixation plates were found dislodged at the time of harvest. These animals were discarded from the study, reducing the number of animals in this group to 25.

#### Bone healing

**Figure 3:**
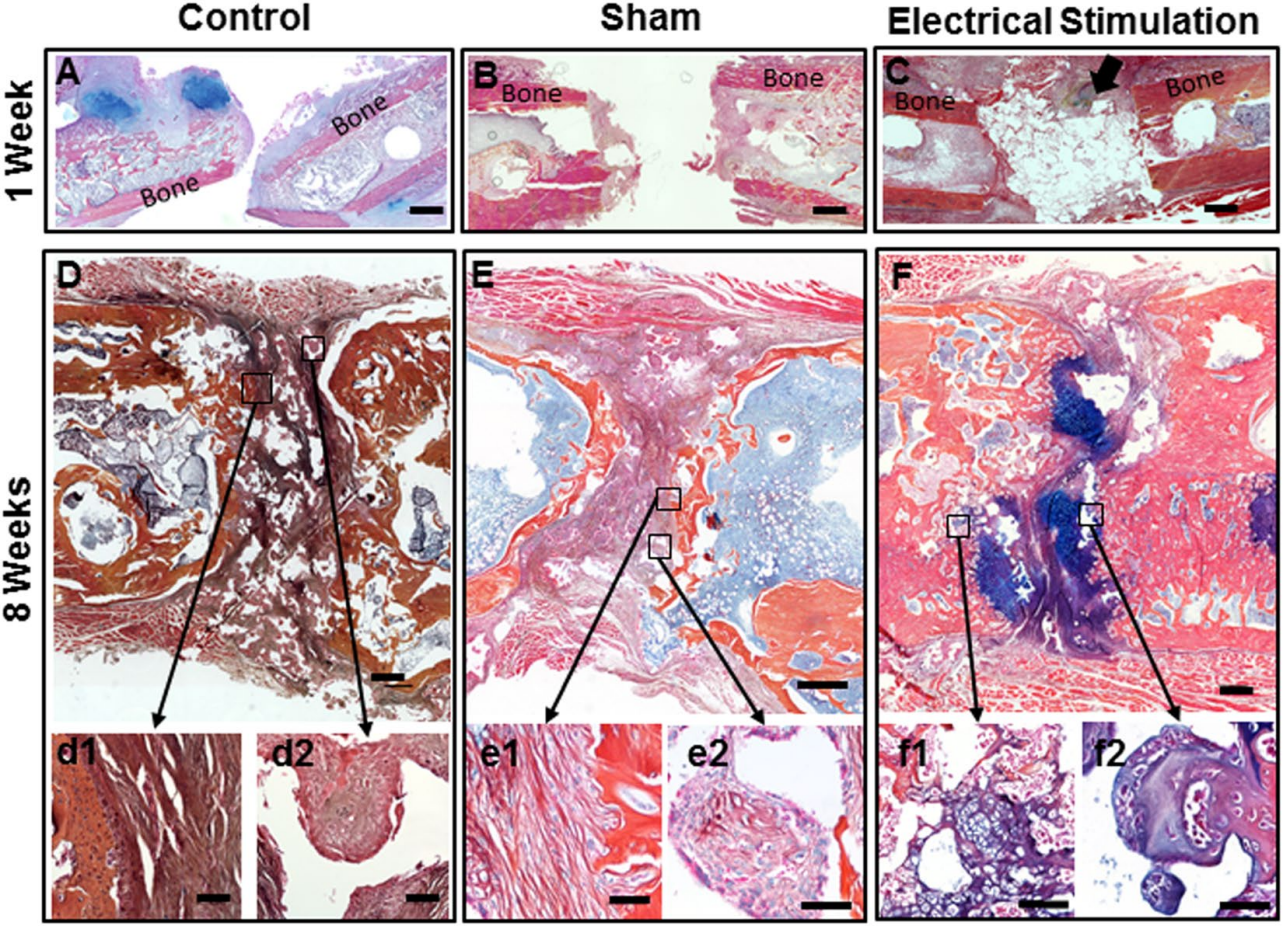
Histological sections of femur defect. After one week post-surgery (**A**) Control, (**B**) Sham, and (**C**) Electrically stimulated groups were stained with Alcian Blue, Orange-G and Hematoxylin. Histological assessment at this time point showed no regeneration in control and sham groups. Arrow shows endochondral ossification center in electrically stimulated group, (scale bar = 1 mm). At eight weeks post-surgery (**D**) Control, (**E**) Sham, and (**F**) Electrically stimulated groups, were assessed with the same histological staining, (Scale bar = 500 μm). Bottom rows are higher magnification images from the location of interest; (d1) fibrous connective tissue within the defect in the control group, (d2) scaffold material covered with fibrous tissue, (e1) fibrous connective tissue within the defect in the sham group, (e2) scaffold material covered with mixed tissues, (f1) cartilaginous and bony tissues within the defect in the experimental group, and (f2) scaffold material covered with cartilaginous and bony tissues, (20×) (scale bar = 50 μm).

Histology, mechanical testing, and gene expression analysis were used to assess bone healing in the defect area at 1 and 8 weeks post-surgery. At 1 week, in 80% of the defects, treated with ES, a soft tissue bridge had formed in the defect gap and endochondral ossification sites were present in 40% of the defects. At the same time point 60% of defects in the control, and 80% in the sham group were not bridged with soft tissue (Fig. 3A–C). By 8 weeks, defects in all 3 groups were filled with non-homogenous combinations of fibrous tissue, hypertrophic cartilage, and bone tissue. Although complete healing was not observed in any of the groups, new bone growth from the proximal and distal bony edges into the center of the defect was evident. In the control group, the middle part of the defect was filled with fibrous connective tissue that covered the scaffold material (Figs 3D,d1,d2, S2). In the sham group fibrous tissue was also present in the defect, however in the middle part of the defect, cartilage- and bone-like tissues were seen covering the scaffold material (Figs 3E,e1,e2, S2). In defects treated with ES, a large area of the defect was replaced with woven bone and hypertrophic cartilage. In these defects there were minimal amounts of fibrous tissue and the scaffold material appeared covered and interconnected with newly formed cartilage and bone (Figs 3F,f1,f2, S2).

**Figure 4:**
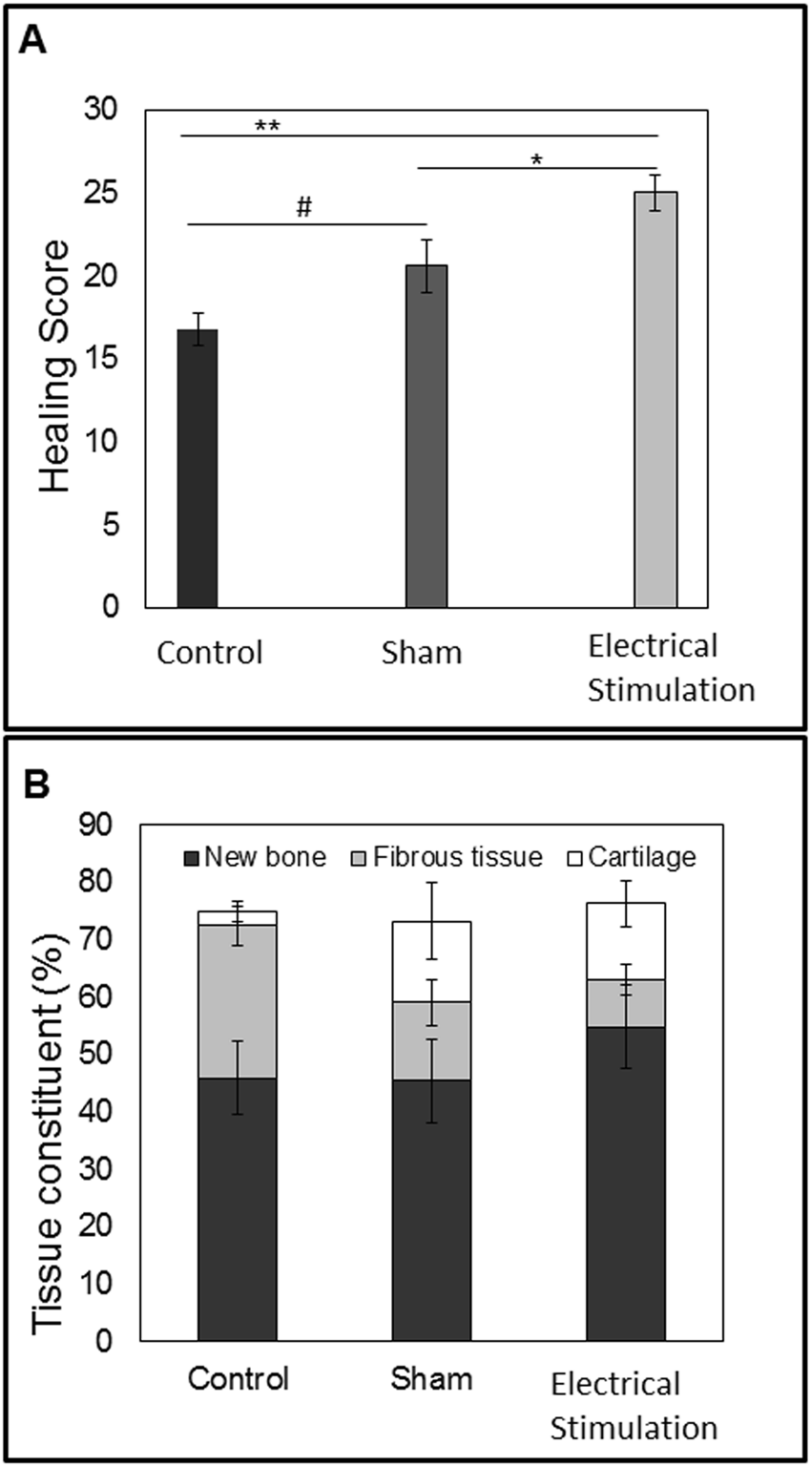
Bone healing score and tissue constituent percentage of the defect. At eight weeks post-surgery, (**A**) Healing scores were calculated using histological image analysis. Data are shown as mean ± SD (n = 5). Experimental group scored significantly higher than control and sham groups, (#p < 0.1; *p < 0.05; **p < 0.01) (**B**) Histomorphometric distribution of newly formed bone, fibrous, and cartilage tissues within the defect, at eight weeks post-surgery. Percentage measured from the whole defect area using ImageJ. Data are shown as mean ± SD (n = 5).

A cumulative histological “bone healing scoring method” was developed and used for quantitative healing evaluation. The distance between bone ends, percentage of bone-like and cartilage-like tissues within the defect, and the presence of fibrous tissue in the defect, were evaluated and scored (Fig. 4A). The healing scores in the ES treated group (25 ± 1.12) were significantly (p < 0.05) higher than those in the sham (20.6 ± 1.57) and control (16.8 ± 0.96) defects. Although the scores in the sham group were higher than in the control group, the difference was not significant. The greatest amount of fibrous tissue and the least amount of new cartilage tissue were seen in the control group. The sham and control group had similar amounts of bone-like tissue. The electrically stimulated defects had the greatest amount of new bone tissue formation and the least amount of fibrous tissue (Fig. 4B).

#### New vessel formation

**Figure 5:**
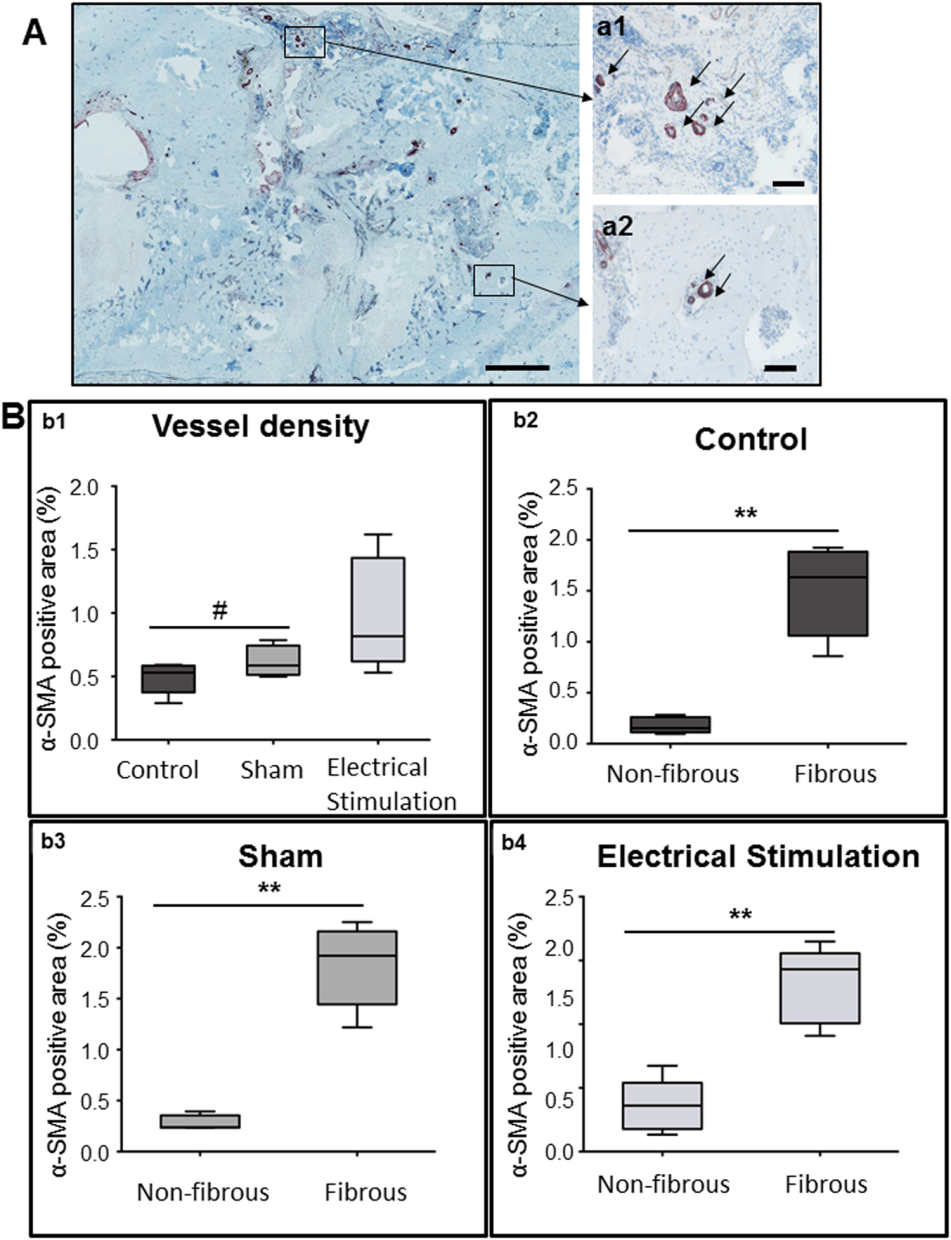
Vascularization in the defect area 8 weeks post-surgery. (**A**) Histological sections of electrically stimulated tissue stained with anti-alpha smooth muscle actin (α-SMA) antibodies (scale bar 1 mm). Higher magnification images of vessels in the fibrous tissue (a1) and in bone tissue (a2) (20×, scale bar 100 μm) (**B**) Vessel density (b1) was calculated for the entire defect area, (#p < 0.1). Vessel density calculated in the fibrous tissue and non-fibrous tissues in controls (b2), sham (b3) and experimental (b4) groups. Data are presented as mean ± SD (n = 5). Vessel density in fibrous tissue was significantly (**p < 0.01) higher than non-fibrous tissue in all groups. Vessel density in fibrous tissue was lower in the experimental than in the sham group (*p < 0.1).

To assess the effect of ES on new vessel formation we stained histological sections of the defects with α-smooth muscle actin antibodies. Vessel density was measured in the entire defect area, and compared to vessel density in fibrous and non-fibrous tissue (Fig. 5). Electrically stimulated defects contained the highest vessel density in the defect in comparison to sham and controls (Fig. 5B,b1). In all groups vessel distribution within the defect was uneven, and the number of vessels in fibrous tissue was significantly (p < 0.05) higher than in non-fibrous tissues (Fig. 5A,B). Overall, sham group samples had the highest vessel density in fibrous tissue while ES treated defects had the highest vessel density in non-fibrous tissue.

#### Bone strength

**Figure 6:**
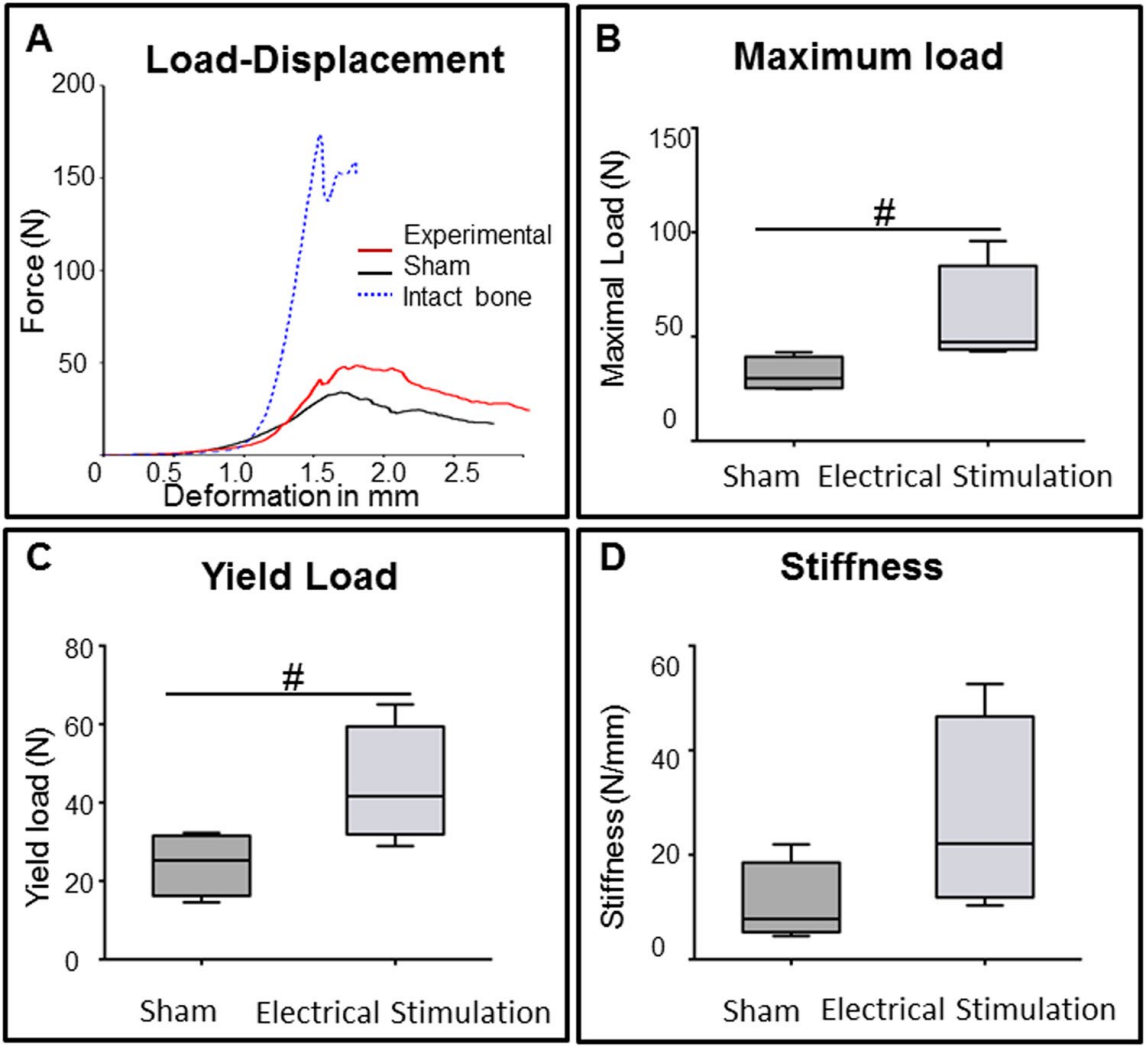
Mechanical properties of newly formed bone in the defect. (**A**) Load displacement diagram of representative femur samples from sham (black), experimental (red) group and intact bone (blue). (**B**) Maximum load before fracture was measured at eight weeks after surgery. Maximum load was higher in experimental compared to sham group (p < 0.1). (**C**) Yield load, in the experimental group was higher than in the sham group (p = 0.058). (**D**) Stiffness was not different between sham and experimental groups (#p < 0.1).

To assess the effect of ES + BTE treatment on the mechanical properties of newly formed bone, 3-point bending tests were performed on the femur defects 8 weeks post-surgery. The load displacement curves of the experimental and sham samples as well as uninjured bone are shown in Fig. 6A. The median maximum load (p < 0.1) and yield load (p = 0.058) were higher in ES treated femurs (Fig. 6B,C). No difference was observed in median stiffness between the groups (Fig. 6D).

#### Gene expression

**Figure 7:**
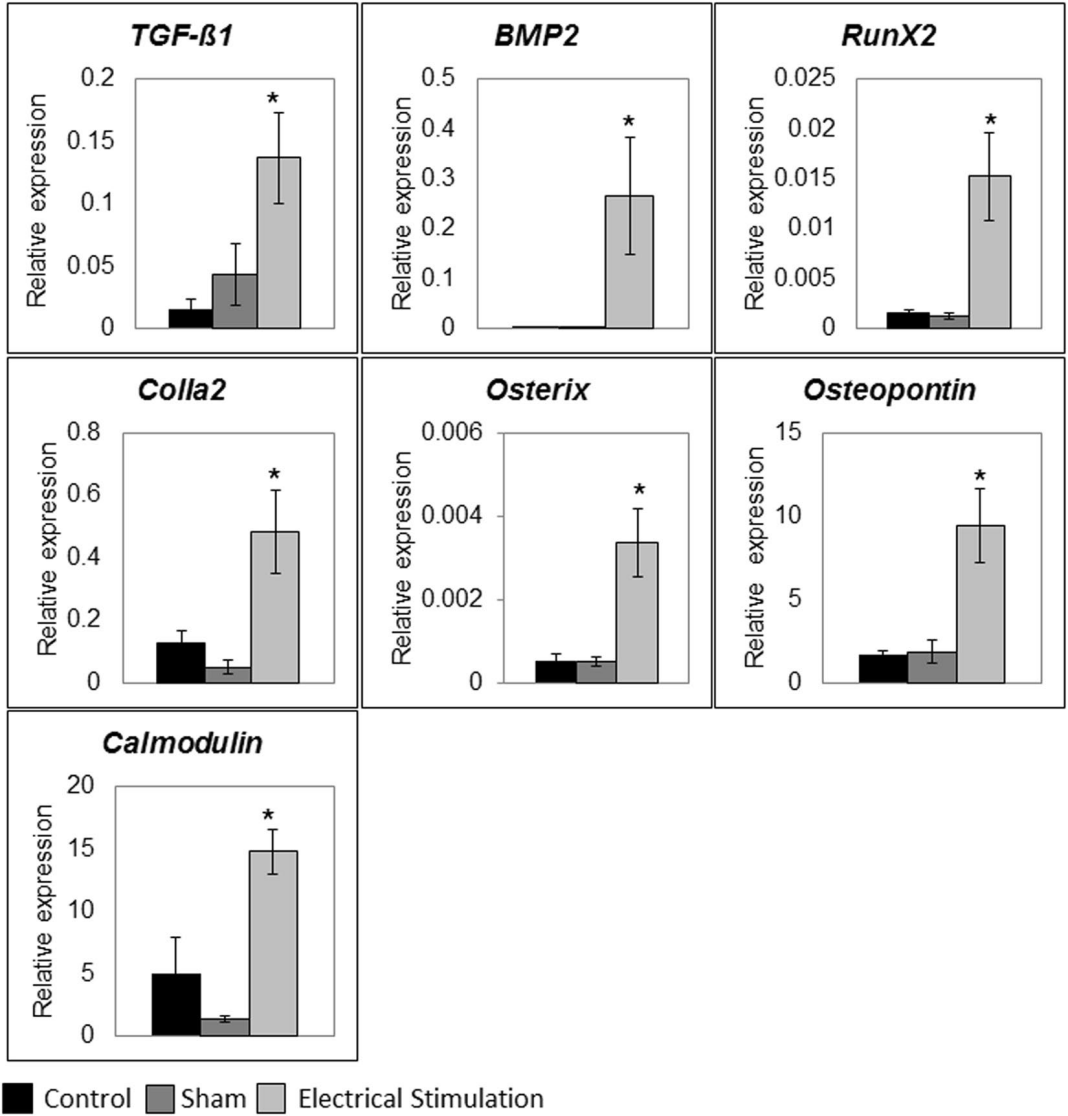
Osteogenic marker gene expression, *in vivo*. At eight weeks post-surgery, osteogenic marker gene expression measured by means of qRT-PCR and normalized to *RPLP1* and *YWHAZ* (housekeeping genes). (**A**) *TGF-ß1*, (**B**) *BMP2*, (**C**) *RunX2*, (**D**) *ColIa2*, (**E**) *Osterix*, (**F**) *Osteopontin* and (**G**) *Calmodulin* expression were significantly higher at eight weeks in the experimental in comparison to sham and control groups. Data are presented as mean ± SD (n = 5), (*p < 0.05).

The effect of ES on bone healing was evaluated via gene expression analysis using qRT-PCR. In the first week ES had no effect on gene expression (data not shown) while at 8 weeks expression of *TGF-ß1* and *Calmodulin* genes were significantly (p < 0.05) increased in the ES treated defects. High amounts of *Bmp2* gene transcript were detected only in ES treated tissues, whereas in sham and controls expression of this gene was negligible. Expression of osteogenic markers *RunX2*, *Osteopontin*, *Col1a2 and Osterix* was also significantly (p < 0.05) higher in the ES-treated compared to sham and control groups (Fig. 7A–G). No effect of ES was detected on the expression of growth factor *VEGF*, and osteogenic marker genes *Osteocalcin* and *ALP* (data not shown).

## Discussion

While in normal circumstances minor fractures heal on their own, large defects tend to overwhelm bone regeneration and the healing processes and require complex and costly treatments. Bone tissue engineering approaches, that combine osteogenic cells, scaffolds, and signaling, hold great promise as an alternative to conventional treatments. Several studies focus on identifying the optimal cell/scaffold/signaling combination with the ultimate goal of reducing healing times and cost, and eliminating unwanted side effects and complications. The effect of ES on bone healing has been studied extensively in animals and humans^19^, however, to our knowledge, combining ES and BTE treatments to enhance healing has not yet been tried. In the present study we wanted to explore the possibility of combining direct current ES and BTE treatments to improve healing. Our findings indicate that ES increases the amount of newly formed bone, and decreases fibrous tissue. We also found that this combination resulted in better vascularization, mechanical properties, and a higher expression of osteogenic marker genes.

In previous studies we showed that exposing AT-MSC in 2D culture to ES significantly enhanced osteogenic differentiation^29^. In the present experiments, in preparation for combining ES and BTE treatments *in vivo*, we sought to reproduce these positive osteogenic effects, however this time combining AT-MSC and scaffold, simulating BTE treatments. Using an *in vitro* setup, we exposed AT-MSC, seeded on ß-TCP scaffold, to ES and found that ES also caused increased osteogenic differentiation in these 3D cell culture conditions. We verified this by measuring activity of the osteogenic marker ALP^38^ and osteogenic marker gene expression and found them both to be significantly elevated. These findings are in accordance with other *in vitro* studies, that found increased ALP activity in MSCs seeded on scaffold in the presence of electrical fields^39–41^.

As it relates to the effect ES has on regulation of osteogenic genes we found mixed results. In our 3D culture setup we observed that while ES caused a significant increase in the expression of *TGF-ß1*, *BMP2*, *Calmodulin* and *Osteopontin* genes, it had no effect on *RunX2*, *ColIa2* and *Osterix* gene expression. While puzzling at first, we also observed these mixed patterns of gene expression in previous experiments where not all osteogenic genes in AT- and BM-derived MSCs responded equally to ES^29^. Further studies are necessary to sort out the reason for these different reactions to ES.

Our histological analysis of BTE treated defects exposed to ES showed a significant improvement in healing. Specifically we found that the BTE treated defects that received ES were replaced with a higher amount of new bone and a lower amount of fibrous tissue. These findings correlated with our mechanical property measurements that showed ES treated defects had stronger flexural resistance than did sham defects. The maximum load in ES treated femurs was around 80 N, while Sham specimens did not support loads higher than 50 N. Flexural strength (Stiffness) of intact rat femurs can vary between 190–250 N/mm, however the direction of the bone during the test may influence these strength values^42^. In the present study, flexural strength of non-injured bones reached between 170 and 190 N/mm and showed stiffer curves when plotted in the graph tension/deformation. Despite not reaching 50% levels of normal uninjured bone, the stiffness values obtained in ES bones were comparable or better than those reported in other^43,44^.

Bone tissue engineering treatment strategies are often hampered by the lack of vascularization within the engineered bone constructs, resulting in poor implant integration and survival (reviewed in^45^). We compared new vessel growth in the bone defect area in all three groups and found that in the ES group vessel density was increased, though this difference was not statistically significant. In previous studies, using a rat limb amputation model, we also saw that ES caused an increase in vascularization^34^, though in this case the increase was much greater. Upon closer analysis of the individual tissues within the defect we found that ES treated defects had the highest vessel density in *non-fibrous* (bone and cartilage) tissue and the lowest in *fibrous* tissue. This finding could not be correlated with other bone healing studies since others do not measure new vessel growth in individual tissues in the defect but instead, the defect as a whole. In dermal wound healing it has been shown that reduced vascularization in fibrous tissue correlates with decreased scar formation^46^. From this we can speculate that ES, by reducing vascularization in fibrous tissue, could have the same effect of reducing fibrous/scar tissue formation in bone defects. Our histological findings correlate with this - ES treated defects contained more bone tissue and less fibrous tissue than did the sham treated defects. This speaks to an overall better healing environment in ES treated defects which likely contributed to the observed increased bone strength measurements mentioned above.

The results of our *in vitro* gene analysis, suggests that osteogenic differentiation of AT-MSCs could have played a key role in the improved bone healing we observed in our ES treated animals. It is known that *TGF-ß* and *BMP2* signaling, promotes osteoprogenitor proliferation and early differentiation to osteoblast-like cells. This signaling pathway is essential in bone formation and homeostasis (reviewed in^47^) and was demonstrated in both our *in vitro* and *in vivo* findings where ES caused up-regulation in the expression of *TGF-ß1* and *BMP2* genes. These genes are responsible for initiating *RunX2* and *Osterix* gene expression, which in turn trigger *ColI* and *Osteopontin* expression and result in promoting osteogenic differentiation^48^. The up regulation of *RunX2*, *ColIa2* and *Osterix* expression we observed in our *in vivo*, but not in our *in vitro* experiments could be explained by a higher complexity and cellular dynamics in the *in vivo* microenvironment. Moreover, the presence of other cell types in the defect area could have been affected by ES and contributed to the observed *RunX2*, *ColIa2* and *Osterix* up regulation. The increase in expression of these genes in the ES group *in vivo* confirms that low voltage ES promotes osteogenesis. Increased expression of *Calmodulin* gene in the ES group, both *in vitro* and *in vivo*, indicates the contribution of calcium/Calmodulin signaling pathways in response to ES. These results correlate with a recent publication by Zhang *et al*.^49^ showing that ES is able to activate ion flux through calcium channels in AT-MSCs and therefore stimulate osteogenic differentiation. In recent studies the crucial role of primary cilium cellular organelle in sensation of electrical field signals and transduction of these signals in osteogenic response was shown in AT-MSCs. The knockdown of cilia structural proteins was shown to suppress ES-induced *BMP2* gene expression and osteogenic differentiation. Function of primary cilia as a crucial calcium-signaling nexus in AT-MSCs during electrical stimulation was suggested as the responsible mechanism^50^.

The present studies focused on the effects of ES on bone forming cells + scaffold, with the ultimate goal of improving outcomes by combining these two treatments. Using *in vitro* and *in vivo* model systems we were able to demonstrate that ES positively affects key bone healing parameters both *in vitro* (changes in cell differentiation and gene expression) and *in vivo* (changes in bone formation, strength and vessel density). While these models and experimental set ups served this purpose well, they are not ideal for measuring the responsible underlying cellular mechanisms. As long term goal in this line of research, ongoing and future studies are focused to determine mechanisms by which ES has this positive effect on bone healing. This knowledge could later optimize results obtained from BTE treatments combined to ES strategies.

In conclusion our *in vitro* studies we showed that exposing AT-MSC + ß-TCP scaffold to ES increases osteogenic gene expression and differentiation. Our *in vivo* experiments showed that exposing AT-MSC + ß-TCP treated defects to ES for 8 weeks resulted in greater amounts of new bone and vessel formation, and less fibrous tissue, increased bone strength and osteogenic gene expression, resulting in overall improved bone healing. Together these findings support the use of ES to improve BTE treatment outcomes.

## Materials and Methods

In *in vitro* studies we first exposed AT-MSC + ß-TCP scaffold to ES and measured osteogenic differentiation. Then, *in vivo*, we treated a rat femur critical size defects with AT-MSC + ß-TCP scaffold, and exposed the treated defect to ES for 1 and 8 weeks, and performed histological, gene expression, and bone strength measurements to determine, if ES improved bone healing.

### *In vitro* studies

To evaluate the effect of ES on BTE treatment we seeded AT-MSC onto ß-TCP scaffold in osteogenic culture medium and exposed the mix to ES (100 mV/mm for 1 h/day). Then at 3, 7, 14, and 21 days we measured cell viability and osteogenic differentiation.

#### AT-MSC Seeding onto β-TCP scaffold

Sprague-Dawley (SD) rat mesenchymal stem cells from adipose tissue (RASMD-01001) were purchased from Cyagen (CA, USA), thawed and cultured as described in detail elsewhere^29^. 2 × 10^5^ AT-MSCs (passage 6) were seeded onto 0.5 mL of β-TCP scaffold granules (0.7–1.4 mm diameter with 60% porosity) (ChronOS, Synthes, Switzerland)- soaked in growing medium for at least 16 hours before seeding, and placed in 6-well plates. In the *in vitro* and *in vivo* experiments the amount of scaffold material used was determined by the amount needed to fill the 5 mm femur defect and the amount of cells used (seeding density) was based on our own previous studies^51–53^. Scaffold + cells were incubated at 37 °C, 5% CO_2_, 5% O_2_ in a humidified incubator for 24 hours. To verify that the AT-MSCs adhered to the 3D scaffold granules, 2 drops of NucBlue fixed cell stain ready probes (Thermo-Fisher Scientific, USA) were added to the cells + ß-TCP scaffold composite and incubated for 5 min at room temperature and then imaged with an Eclipse Ti fluorescent microscope (Nikon instruments, Japan). After 24 hours (day 0) growth medium was exchanged with osteogenic medium (OM) supplemented with 10^−7^ M dexamethasone, 10 mM β-glycerophosphate, and 0.05 mM ascorbic acid-2-phosphate (Sigma-Aldrich, Germany).

#### Electrical stimulation of the cells+ß-TCP scaffold in culture

One day after seeding the cells + scaffold were exposed to ES as described in detail elsewhere^29^. Briefly, cells + scaffold were exposed to 100 mV/mm for 1 h/day in a custom-made ES cell culture device^28^. Medium was changed every 2–3 days. Controls (no electrical stimulation) were treated the same and kept in the same conditions.

#### Cell AT-MSC metabolic activity (viability) measurements

Cytotoxic effects of ES in osteogenic culture conditions was assessed using MTT assay (Cell proliferation Kit I MTT, Roche, CH). At day 3, 7, 14, and 21 ß-TCP scaffold + cells were washed twice with PBS solution (Sigma, Germany) and transferred into a new well. MTT assay was performed according to the manufacturer’s protocol. Data are shown as fold change relative to that of day 0.

#### Osteogenic differentiation measurements

Alkaline Phosphates (ALP) activity was used to measure osteogenic differentiation. ß-TCP scaffold + cells were treated with lysis buffer (400 mM potassium phosphate buffer, 0.01 mM EDTA, 2% triton X-100, pH = 7), and 3 freeze-thaw cycles followed by sonication (45 KHz, 10 minutes, + 4 °C). Cell lysates were collected in fresh micro tubes and processed for ALP activity measurements. ALP assay was performed on all groups at days 3, 7, 14, and 21 according the manufacture’s protocol (SensoLyte pNPP Alkaline Phosphatase Detection kit, Anaspec Inc, CA, USA). Absorbance was measured at 405 nm using Infinite 200PRO NanoQuant plate reader (Tecan, Germany). ALP measurements were normalized against cell number.

#### Osteogenic marker gene expression analysis

To measure the effect of ES on osteogenic gene expression, total RNA from cells was isolated using Aurum Total RNA mini Kit (BioRad, Germany) following the manufacturer’s instructions. The quality and quantity of RNA were measured using gel electrophoresis and an Infinite 200PRO NanoQuant device (Tecan, München, Germany), respectively. DNase-treated RNA samples were reverse transcribed using iScript Select cDNA Synthesis Kit (Bio-Rad, Germany) according to the manufacturer’s instructions. The qRT-PCR reaction was performed using cDNA equivalent of 10 ng RNA and the SsoAdvanced Universal SYBR Green Supermix (BioRad, Germany). All samples were amplified in duplicates using a CFX96 Touch Real Time PCR Detection System (BioRad, Germany) with rat gene specific primers (Sigma Aldrich, Germany), described in Supplementary Table S1. Ribosomal protein P1 (*RPLP1*) and tyrosine 3-monooxygenase/tryptophan 5-monooxygenase activation protein zeta (*YWHAZ*) were both used as reference genes in each experiment^54^. A melting curve analysis was applied to ensure the specificity of the PCR procedure. Amplification products were also analyzed by gel-electrophoresis. Relative quantification of messenger RNA (mRNA) levels of the target genes was analyzed using the comparative C_T_ (threshold cycle values) method (2^−ΔCt^)^55^. The results are presented as relative quantification (RQ), which is expression fold change compared to the housekeeping genes. Three samples were analyzed for each group and mean value and standard deviation were calculated for further analysis.

### *In vivo* studies

All animal experiments were performed according to institutional guidelines and approved (Project No. FU1030) by our University’s animal care and oversight committee (Regierungspräsidium Darmstadt, Veterinärdezernat, Wilhelminenstraße 1–3) according to German law. Eighty one, nine-week-old male Sprague Dawley (SD) rats (Charles River Labs Int., Germany) were randomly allocated in three groups that received: (1) Electrical stimulation + β-TCP scaffold + AT-MSC (Experimental Group; n = 27), (2) No electrical stimulation (disabled ES device) + β-TCP scaffold + AT-MSC (Sham Group; n = 27), and (3) β-TCP scaffold alone (Control Group; n = 27) (Supplementary Table S2).

#### Rat femur critical size defect creation

Under general anesthesia (Ketamine, 100 mg/kg and xylazine hydrochloride, 10 mg/kg, IP), the right hind limbs of rats were shaved, cleaned with antiseptic fluid and a 3 cm longitudinal dermal incision was made over the femur. The superficial fascia was incised and the tensor fascia lata, biceps femoris, and vastus lateralis muscles were elevated from the greater trochanter exposing the lateral aspect of the femur. A five-hole plate (Apothecaries’ Sundries Mfg. Co, India) was fixed to the lateral aspect of the femur with 2 proximal and two distal cortical screws. Once the plate was secured in place a 5 mm long defect was created on the femur shaft beneath the mid-point of the plate using a 0.22 mm gigli wire saw (RISystem, Switzerland).

#### Electrical stimulation

**Figure 8:**
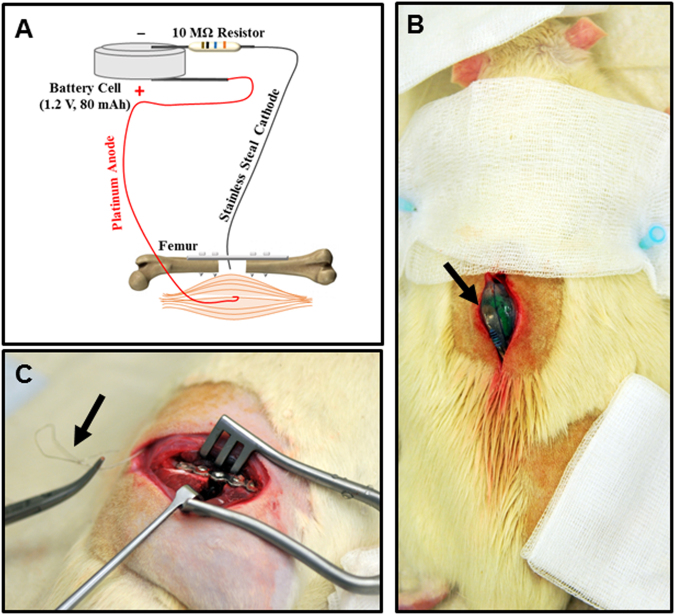
*In vivo* rat model and electrical stimulation set-up. (**A**) Schematic shows the electrical stimulation device and the location of the anode and cathode in the defect. Device consists of a 1.2 V battery, 10 MΩ resistor, stainless steel cathode (shown in black) and platinum anode (shown in red). Cathode located within the defect, and the anode located in muscle tissue close to the defect area. (**B**) Dorsal view of rat with surgical incision and implanted device (arrow). (**C**) Surgical incision exposing right femur defect stabilized with stainless steel plate and screws, and electrodes (arrow) tunneled from the device to the defect area.

Electrical stimulation was delivered to femur defects using a custom-made device, consisting of a Nickel-metal hydride (NiMH) cell battery (1,2 V, 80Mah, Emmerich, Germany), 10 MΩ resistor, 10 cm platinum wire electrode (Anode) (0.125 mm, 99.99%, PTFE isolated, Goodfellow, Germany) and 10 cm stainless steel wire electrode (Cathode) (0.228 mm, type 316, PTFE isolated seven strand wire, Medwire, USA), which delivers 0.1–0.2 µA of direct current (Fig. 8A). The electrode-resistor soldering was reinforced with quick drying, 2-component epoxy resin glue and the union was completely encapsulated and isolated in medical grade silicone (RTV-coating, Dow Corning, USA), leaving only the ends of the electrodes exposed. In order to prevent device breakage, the platinum and steel wires were covered with flexible silicon tubing. Two small 3–0 Vicryl suture loops were placed in the devices’ silicone cover on either side serving as handles to hold them in place in the animals. Prior to implantation ES devices were sterilized in 70% Ethanol for 1 hour, exposed to UV light for 1 hour and rinsed with sterile PBS solution (Sigma-Aldrich, Germany). In the sham group a 2.5 cm long piece of stainless steel wire (identical to the cathode electrode in ES device) served as the “disabled” device.

In ES treated animals, after creating the defect, a 3 cm incision was made on the back of the animal, and a subcutaneous pocket was gently dissected to accommodate the battery part of the ES device, measuring, 2 cm diameter × 1 cm height. Then the ES device was placed in the subcutaneous pocket (Fig. 8B), the stainless steel and platinum electrodes were tunneled subcutaneously to the bone defect area with a curved Halstead Mosquito Forceps (Fig. 8C). Then the ES device was fixed in place with 2 suture loops in the adjacent fascia. The distal end of stainless steel electrode (cathode) was fixed in the middle of the bone defect while the distal end of platinum electrode (anode) was secured in muscle tissue in the vicinity of the defect (Fig. 8A). In sham animals only a small segment of stainless steel wire was fixed in the middle of the bone defect. After defects received their respective treatments the wound was irrigated with sterile saline, the fascia was re-approximated and sutured (3–0 Vicryl; Ethicon) and the skin was closed with continuous intradermal sutures (4–0 Prolene; Ethicon).

#### AT-MSC culture and seeding onto β-TCP scaffold material

AT-MSCs were cultured and seeded onto β-TCP scaffold using the same method as described above in the *in vitro* experiments. For each animal 2 × 10^5^ AT-MSCs (passage 6) were seeded onto 0.5 mL of β-TCP scaffold granules (0.7–1.4 mm diameter with 60% porosity) saturated with medium. Seeded cells (experimental and sham groups) or empty scaffold (control group) were incubated at 37 °C, 5% CO_2_, 5% O_2_ in a humidified incubator one hour prior to being transplanted into the femur defect and were transported to the animal facility.

#### Histological assessment of bone healing

Histological assessment of defect tissues was performed at 1 and 8 weeks post-surgery. Animals were euthanized using CO_2_ inhalation and their femurs were dissected free and examined macro- and microscopically for signs of infection or tumors. Plates and screws were removed and femurs were fixed in Zinc-Formal-Fixx (Thermo Scientific, USA) for 24 hours, decalcified in 10% EDTA/TRIS-HCl (pH 7.4) for 14 days, and embedded in paraffin for subsequent histomorphometric analysis. Tissue sections (5–7 µm) were taken parallel to the long axis of the femur and stained for bone healing assessment with Alcian Blue-Orange G-Hematoxilin-Eosin according to the protocol published elsewhere^56^. Images of the sections were captured using light microscopy (Ti-E, Nikon GmbH, Germany) and analyzed with NIS-Elements software (NIS-Elements 4.4, Nikon GmbH, Germany). Samples collected at week 1 post-surgery were evaluated for defect area soft tissue bridging. In order to assess healing, samples collected at 8 weeks post-surgery were scored based on the Lane and Sandhu scoring system^57^. We measured the defect area not yet healed (remaining bone defect size) and the area of newly formed bone, cartilage, and fibrous tissue (in µm^2^). These area measurements were normalized to the size of the original defect area (in %) and assigned a score from 1 to 10 for each of 4 separate parameters (bone defect area, cartilage area, bone area, and fibrous tissue area) (Supplementary Table S3). These healing scores were derived from histological assessment of 5 animals per group and 2–3 samples per animal.

#### New vessel formation measurements

Paraffin embedded sections were incubated with monoclonal mouse anti α-smooth muscle actin antibody (1:200, ABCAM, Germany). An isotype-identical (IgGa2, $\underline{{\rm{k}}}$) non-specific mouse antibody served as a negative control (BioLegend, USA). For signal detection, an En Vision + System-HRP (AEC) kit (Dako, Germany) was used. Finally, Hematoxylin counterstain was performed. Quantitative evaluation of vessel staining was performed on standardized images of histological sections using light microscopy and ImageJ 1.5i analysis software. Assessments were performed in blinded specimens examined in random order.

#### Bone biomechanical measurements

For biomechanical tests, bone specimens collected at 8 weeks post-surgery, were wrapped with gauze dampened with physiologic saline and then stored in 70% ethanol at 4 °C. Biomechanical testing was performed using a destructive three-point bending test with a universal material testing machine (Prüfmaschine Zwicki-Line Z5.0; Zwick/Roell, Ulm, Germany) as described elsewhere^44^. Briefly, the femur was placed on two rounded bars separated by a distance of 20 mm, so that the bone defect zone was halfway between the bars. The “bending until failure” procedure was performed by lowering one bar onto the femur using a constant deflection speed of 0.1 mm/sec, recording load, and deflection continuously. From the data, a load versus displacement graph was generated and maximal load, yield load, stiffness, were calculated (TestXpert-II software) and used to calculate median values for each group (n = 5). These values were then statistically analyzed.

#### Gene expression analysis

Gene expression analysis was performed on defect tissues collected at 1 and 8 weeks post-surgery, snap frozen in liquid nitrogen and stored at −80 °C. Total RNA from frozen tissue was isolated using TRI-Reagent (Sigma-Aldrich, Germany) following the manufacturer’s instructions. Genomic DNA contamination was removed through digestion using RNase-free DNaseI following the manufacture’s provided protocol (New England BioLabs GmbH, Germany). DNase-treated RNA samples were processed for qRT-PCR. The qRT-PCR reaction was performed using cDNA equivalent of 10 ng RNA as described in *in vitro* section. Relative quantification of mRNA levels of target genes was performed as described in *in vitro* section. Five samples were analyzed for each group and the mean value and standard deviation were calculated for further analysis.

#### Statistical analysis

All *in vitro* experiments were done in triplicates. In the *in vivo* studies a minimum of five animals per group per analysis were used. The qRT-PCR data are presented as mean ± SD and significance level was set at p < 0.05. For vascularization analysis and biomechanical testing results are presented as box-plots of the median in figures, 25%, and 75% quartiles ((M (25%q/75%q)). Nonparametric Kruskal–Wallis test and multiple Conover-Iman test were consequently used, and a Bonferroni-Holm corrected p < 0.05 was used to indicate statistical significance. Statistics were calculated using the software *Bias* 10.03 (Epsilon-Verlag, Darmstadt, Germany).

## Electronic supplementary material

Supplementary Information
