# Supplementary material for: Combining electrical stimulation and tissue engineering to treat large bone defects in a rat model
Source: Sci Rep. 2018 Apr 20;8:6307. doi: 10.1038/s41598-018-24892-0 (PMC5910383; doi:10.1038/s41598-018-24892-0)
Supplement: Supplementary file 1 — Supplementary Information [file 41598_2018_24892_MOESM1_ESM.doc]

**Supplementary Information**

**Combining electrical stimulation and tissue engineering to treat large bone defects in a rat model**

Liudmila Leppik^1+^, Han Zhihua^1+^, Sahba Mobini^1+, 2^, Vishnu Thottakkattumana Parameswaran^1, 3^, Maria Eischen-Loges^1^, Andrei Slavici^1, 4^, Judith Helbing1^, 5^, Lukas Pindur^1, 6^, Karla M. C. Oliveira^1^, Mit B. Bhavsar^1^, Lukasz Hudak^1^, Dirk Henrich^7^, John H. Barker^1*^


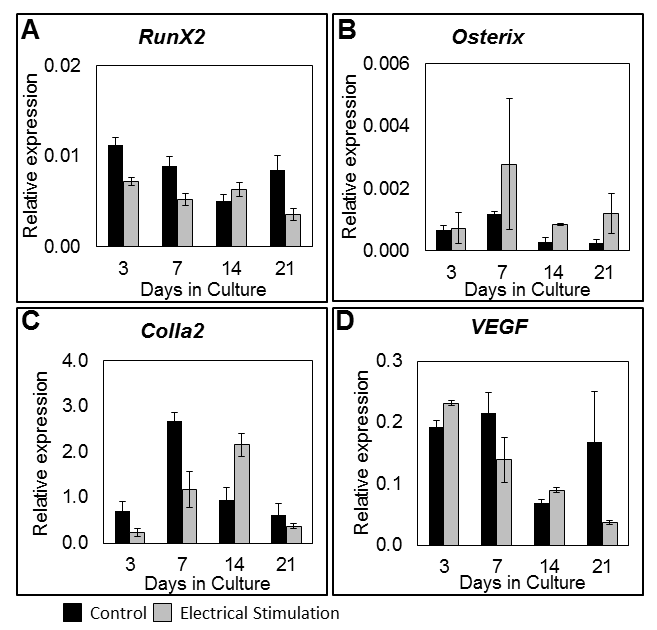


**Supplementary figure S1. Effect of electrical stimulation on osteogenic marker gene expression, *in vitro*.** Additional measurements of osteogenic marker gene expression were performed by means of qRT-PCR and normalized to *RPLP1* and *YWHAZ* (housekeeping genes). There were no significant difference in the expression of **A)** *RunX2,* **B)** *Osterix, C***)** *ColIa2* and **D)** *VEGF* between the control and electrically stimulated groups. Data are presented as mean + SD, (n=3).

**
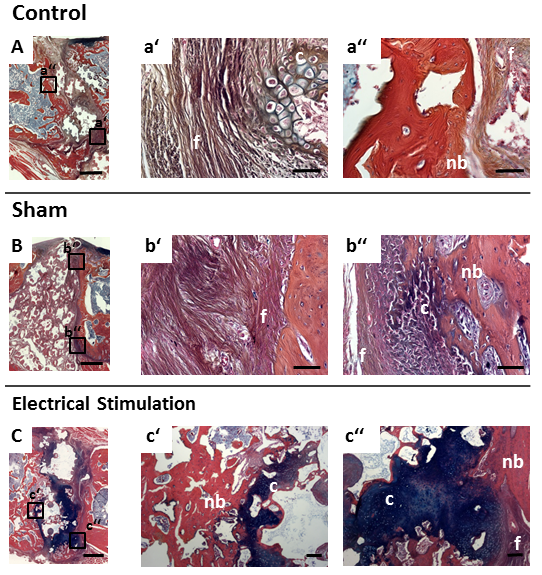
**

**Supplementary figure S2. Representative histological sections of femur defect.** Eight weeks after defect creation **A)** Control, **B)** Sham, and **C)** Electrically stimulated bone defects were stained with Alcian Blue, Orange-G and Hematoxylin, (4X) (scale bar=500 μm). In the high magnification images the different tissue types are indicated with the following letters “nb” = new bone, = nb, “c” = cartilage, and “f” = fibrous connective tissue. Two high magnification (20X) regions of interest “boxes” in the control - a’ & a’’, Sham - b’ & b’’, and ES - c’ & c’’ tissues appear to the right. (scale bar = 100 μm).

**Supplementary Table S1.** qRT-PCR primer sequences.

| Gene | Forward Primer (5’-3’) | Reverse Primer (5’-3’) |
| --- | --- | --- |
| *ALP* | CCTGGACCTCATCAGCATTT | AGGGAAGGGTCAGTCAGGTT |
| *BMP2* | AACGAGAAAAGCGTCAAGCC | CCAGTCATTCCACCCCACA |
| *Calmodulin* | TTTGACAAGGATGGCAATGGCT | TGTTAGCTTTTCCCCGAGGT |
| *Col1a2* | TTCCCGGTGAATTCGGTCT | ACCTCGGATTCCAATAGGACCAG |
| *Osteocalcin* | GGAGGGCAGTAAGGTGGTGA | GAAGCCAATGTGGTCCGC |
| *Osteopontin* | GATGAACAGTATCCCGATGCC | TCCAGCTGACTTGACTCATGG |
| *Osterix* | CTGGGAAAAGGAGGCACAAAG | GGGTGGGTAGTCATTGGCATAG |
| *RPLP1* | GCATCTACTCCGCCCTCATC | GCATCTACTCCGCCCTCATC |
| *RunX2** | * | * |
| *TGF- β1* | CTGCTGACCCCCACTGATAC | AGCCCTGTATTCCGTCTCCT |
| *VEGF* | CTGCCTGGAAGAATCAGGAG | GAGGAGGAGGAGCCATTACC |
| *YWHAZ* | GATGAAGCCATTGCTGAACTTG | GTCTCCTTGGGTATCCGATGTC |
| *ALP:* Alkaline phosphatase*; BMP-2:* Bone morphogenic protein*-2; Col1a2:* collagen type I alpha 2 chain*; RPLP1:* Ribosomal protein P1*; RunX2:* Runt-related transcription factor 2*; TGF-β1:* transforming growth factor- β1*; VEGF:* vascular endothelial growth factor*; YWHAZ:* tyrosine 3-monooxygenase/tryptophan 5-monooxygenase activation protein zeta*. *- RunX2* primers were purchased from Qiagen, Germany (330523). | | |

**Supplementary Table S2. *In vivo* group setup.**

| ***Group*** | ***Treatment*** | ***Weeks post-surgery*** | ***Number of animals*** | ***Analyses (n=5)*** |
| --- | --- | --- | --- | --- |
| Electrical Stimulation | Electrical stimulation  β-TCP scaffold  AT-MSC | 1 | 10 | Histology, gene expression |
|  |  | 8 | 17 | Histology, bone mechanical test, gene expression |
| Sham | Disabled device  β-TCP scaffold  AT-MSC | 1 | 10 | Histology, gene expression |
|  |  | 8 | 17 | Histology, bone mechanical test, gene expression |
| Control | - Electrical stimulation  +β-TCP scaffold  - AT-MSC | 1 | 10 | Histology, gene expression |
|  |  | 8 | 17 | Histology, bone mechanical test, gene expression |

**Supplementary Table S3.** Parameters assessed to calculate healing score.

|  | Healing Score | | | |
| --- | --- | --- | --- | --- |
|  | Bone defect size | Bone | Cartilage | Fibrous tissue |
| 0-10% | 10 | 1 | 1 | 10 |
| 11-20% | 9 | 2 | 2 | 9 |
| 21-30% | 8 | 3 | 3 | 8 |
| 31-40% | 7 | 4 | 4 | 7 |
| 41-50% | 6 | 5 | 5 | 6 |
| 51-60% | 5 | 6 | 6 | 5 |
| 61-70% | 4 | 7 | 7 | 4 |
| 71-80% | 3 | 8 | 8 | 3 |
| 81-90% | 2 | 9 | 9 | 2 |
| 91-100% | 1 | 10 | 10 | 1 |
